# Neighborhood Resources to Support Healthy Diets and Physical Activity Among US Military Veterans

**DOI:** 10.5888/pcd14.160590

**Published:** 2017-11-09

**Authors:** Elizabeth Tarlov, Shannon N. Zenk, Stephen A. Matthews, Lisa M. Powell, Kelly K. Jones, Sandy Slater, Coady Wing

**Affiliations:** 1 College of Nursing, University of Illinois at Chicago, Chicago, Illinois; 2 Center of Innovation for Complex Chronic Healthcare, Edward Hines, Jr. VA Hospital, Hines, Illinois; 3 Department of Sociology, Anthropology, and Demography, The Pennsylvania State University, University Park, Pennsylvania; 4 School of Public Health, University of Illinois at Chicago, Chicago, Illinois; 5 School of Public and Environmental Affairs, Indiana University–Bloomington, Bloomington, Indiana

## Abstract

**Introduction:**

Among the nearly 21 million military veterans living in the United States, 64.0% of women and 76.1% of men are overweight or obese, higher rates than in the civilian population (56.9% of women and 69.9% of men). Attributes of the residential environment are linked to obesity. The objective of this study was to characterize the residential environments of the US veteran population with respect to availability of food and recreational venues.

**Methods:**

We used American Community Survey data to determine the concentration of veterans (the percentage of veterans among the adult population) in all continental US census tracts in 2013, and we used proprietary data to construct measures of availability of food and recreational venues per census tract. Using descriptive statistics and ordinary least-squares regression, we examined associations between the concentration of veterans per census tract and those residential environmental features.

**Results:**

In census tracts with high concentrations of veterans, residents had, on average, 0.5 (interquartile range, 0–0.8) supermarkets within a 1-mile radius, while residents in census tracts with low concentrations of veterans had 3.2 (interquartile range, 0.6–3.7) supermarkets. Patterns were similar for grocery and convenience stores, fast food restaurants, parks, and commercial fitness facilities. In adjusted analyses controlling for census-tract–level covariates, veteran concentration remained strongly negatively associated with availability of those food and recreational venues. In nonmetropolitan tracts, adjusted associations were greatly attenuated and even positive.

**Conclusion:**

Where veterans live is strongly associated with availability of food outlets providing healthy (and unhealthy) foods and with recreational venues, raising questions about the contributions of veterans’ residential environments to their high obesity rates. Additional research is needed to address those questions.

## Introduction

Among the nearly 21 million military veterans living in the United States, approximately 64.0% of women and 76.1% of men are overweight or obese (1,2). These rates are derived from self-reported heights and weights provided by veteran respondents to the Behavioral Risk Factor Surveillance Survey (BRFSS). Nonveteran BRFSS respondents have lower rates of overweight and obesity: 56.9% for women and 69.9% for men (1,2). Facilitating healthy diets, physical activity, and weight management in the veteran population is an important public health challenge.

Veterans’ residential patterns differ from those of the general population. Some states are home to a disproportionate number of veterans, partly because veterans tend to cluster in areas near military installations and in rural areas (3,4). Food and recreational venues such as supermarkets, fast food restaurants, parks, and fitness facilities, which have been linked to obesity risk, are unevenly distributed across areas (5–9). Race and socioeconomic status — factors known to be correlated with the availability of food and recreational venues — are correlated with veteran status. For example, compared with the population of all US adults, the veteran population has lower percentages of African American and Latino people, higher rates of high school graduation, and lower rates of college graduation (3,10). Employed veterans typically earn more than the median income of employed workers in the overall population. However, veterans are much more likely to be out of the labor force entirely (11,12). The combination of veteran characteristics makes it hard to predict whether veterans as a group are more likely or less likely to have access to food and recreational venues close to home. Our objective was to characterize the residential environments of the US veteran population with respect to the availability of food and recreational venues. Knowing about spatial access of the veteran population to these residential environmental attributes may reveal potential contributors to, and opportunities to address, the high rates of overweight and obesity among the veteran population.

## Methods

We compared the availability of several types of food outlets, commercial fitness facilities, and parks across geographic areas with various concentrations of veterans. We conceptualized these residential environmental attributes as both resources (supermarkets and grocery stores, parks, and commercial fitness facilities) and as barriers (convenience stores and fast food restaurants) to the adoption and maintenance of healthy behaviors. The sample comprised all 2,013 census tracts in the continental United States with an adult population greater than zero.

### Measures

**Concentration of veterans.** We used 5-year estimates from the American Community Survey 2009–2013, including data on self-reported veteran status, to determine the percentage of veterans among the adult population in each census tract (13). We categorized concentrations into quartiles: low (0%–6.0%), mid-low (6.0%–8.8%), mid-high (8.8%–11.5%), and high (11.5%–100%).

**Availability of food and recreational venues.** We obtained data on the locations of retail food outlets from InfoUSA (supermarkets, grocery stores, convenience stores) and Dun & Bradstreet (fast food restaurants) databases. Data on the locations of recreational venues were obtained from InfoUSA (commercial fitness facilities) and Navteq and TeleAtlas (parks) databases. Details on data collection are described elsewhere (14,15). To construct census-tract measures, we divided the continental United States into cells measuring 30 m by 30 m (approximately 8.9 billion cells in all) and identified the cells whose centroids (geometric centers) were contained within the boundaries of each census tract, using ArcGIS Desktop Release 10.2.1 (Esri). We counted the number of each type of food outlet and recreational venue within a 1-mile radius of each centroid. The availability of each food or recreational venue in the census tract is the average count across all cells in the census tract. This approach allowed us to characterize the availability of food and recreational venues across the census tract rather than just for its centroid. It also avoided characterizing availability only within census-tract boundaries, recognizing that residents’ access is not confined to venues located within the census tract.

To measure relative availability of venues, we calculated a location quotient for each measure. A location quotient — used in geography, economics, and health services research — is the ratio of the availability of an environmental venue in one location relative to its availability across all locations in a sample (16,17); the greater the location quotient, the greater the relative availability. The numerator is the population-weighted mean value of the venue (for example, counts of supermarkets within 1 mile) for census tracts in a given quartile, and the denominator is the population-weighted mean across all census tracts in the continental United States. That is, the location quotient expresses the level of a variable in one geographic area as a percentage of the national average.

**Census-tract characteristics.** To control for other census-tract characteristics, we used American Community Survey 5-year estimates of census-tract sociodemographic characteristics (age, race/ethnicity, population density), economic characteristics (median household income), and geographic characteristics (census division) (13). As a measure of urbanicity, we used the National Center for Health Statistics’ 2013 Urban–Rural Classification Scheme for Counties (18) and classified each census tract according to its county location. This classification scheme has 6 categories, from the most urban (large central metropolitan) to the most rural (noncore). For some analyses, we grouped counties as metropolitan or nonmetropolitan and defined the nonmetropolitan group as including the 2 most rural categories (noncore and micropolitan). Finally, to account for potential access to food and recreational venues associated with military installations to which many veterans have access and which may not be available in our data, we used location data from the Defense Installations Spatial Data Infrastructure Program (19) to calculate distance to the nearest military installation as the straight-line distance from the census-tract centroid to the closest boundary of the installation.

### Statistical analysis

We compared the availability of food and recreational venues in census tracts grouped according to quartiles of concentration of veterans, using means, standard deviations (SDs), and interquartile ranges (IQRs); we used 1-way analysis of variance for continuous variables and χ^2 ^tests for categorical variables. We also examined location quotients graphically. Finally, to gain further insight into relationships between veterans’ residential locations and their potential access to dietary and physical activity–related resources and barriers, we fit ordinary least-squares regression models of availability measures as a function of veteran concentration while controlling for census-tract–level covariates. We estimated 2 models for 2 groups of tracts: all census tracts and census tracts located in nonmetropolitan areas. Model 1 included only the sociodemographic, economic, and geographic characteristics of census tracts. Model 2 added concentration of veterans and a measure of distance to the nearest military installation. Stata version 13.1 (StataCorp LLC) was used for all analyses.

## Results

The mean concentration of veterans, by census tract, ranged from 0% to 100%; the mean concentration of veterans among all tracts was 9.1% (SD, 4.7%) (Table 1). In census tracts with a low concentration of veterans, 3.8% (SD, 1.6%) of the population on average were veterans, while in high-concentration tracts, 14.9% (SD, 4.5%) were veterans. Most sociodemographic characteristics of census-tract populations differed across quartiles of veteran concentration. Census tracts with a high concentration of veterans had higher percentages of adults aged older than 65, non-Hispanic whites, and high school graduates than had census tracts with low, mid-low, and mid-high concentrations of veterans, but they had lower percentages of college graduates. In contrast, median household income varied little across quartiles. One-quarter (approximately 25%) of high-concentration census tracts, but only 5.0% of low-concentration census tracts, were located in nonmetropolitan areas. 

**Table 1:** Sociodemographic Characteristics of Census-Tract Populations and Geographic Characteristics of Census Tracts, by Concentration of Veterans, Continental US Census Tracts, 2009–2013^a^

Variable	All Tracts (N = 71,899)	Concentration of Veterans by Quartile^a^			
		Low (N = 17,972)	Mid-Low (N = 17,980)	Mid-High (N = 17,974)	High (N = 17,973)
**Population, mean (SD), n**	3,268 (1,477)	3,280 (1,559)	3,340 (1,489)	3,295 (1,412)	3,156 (1,436)
**Concentration of veterans,^a^ mean (SD), % [IQR]**	9.1 (4.7) [6.0–11.5]	3.8 (1.6) [2.6–5.1]	7.5 (0.8) [6.8–8.2]	10.1 (0.8) [9.5–10.8]	14.9 (4.5) [12.4–15.8]
**Adults aged >65 y, mean (SD), %**	18.1 (8.6)	12.8 (6.4)	16.8 (6.0)	19.4 (6.2)	23.3 (10.9)
**Race/ethnicity, mean (SD), %**					
Non-Hispanic white	63.7 (30.2)	39.9 (30.5)	63.2 (28.1)	74.1 (24.4)	77.6 (21.3)
Non-Hispanic black	13.5 (22.1)	17.9 (25.3)	15.4 (23.9)	11.4 (20.3)	9.3 (17.0)
Non-Hispanic Asian	4.3 (8.3)	8.7 (12.9)	4.3 (7.0)	2.3 (4.2)	1.9 (3.4)
Non-Hispanic other	3.0 (5.1)	3.0 (6.1)	3.0 (5.3)	2.9 (4.8)	3.1 (4.1)
Hispanic	15.5 (21.2)	30.5 (29.2)	14.1 (17.8)	9.3 (13.2)	8.1 (11.2)
**Education, mean (SD), %**					
<High school diploma or GED	14.7 (11.6)	21.2 (16.1)	14.1 (10.4)	12.6 (8.0)	11.0 (6.7)
High school diploma, no bachelor’s degree	57.5 (14.0)	48.6 (14.5)	56.2 (13.6)	61.5 (11.7)	63.8 (10.8)
Bachelor’s degree or more	27.7 (18.6)	30.2 (23.0)	29.7 (19.5)	25.9 (15.9)	25.2 (14.1)
**Median household income, mean (SD), in thousands, $**	56.4 (28.3)	54.1 (32.8)	60.2 (31.6)	56.4 (24.8)	54.9 (22.0)
**Population per km^2^, mean (SD), in thousands, n**	5.1 (11.5)	13.1 (19.8)	3.7 (5.9)	2.2 (3.3)	1.6 (2.1)
**Distance to military installation,^b^ mean (SD), km**	69.3 (77.0)	57.3 (72.2)	71.6 (78.6)	79.2 (79.7)	69.1 (75.7)
**Urbanicity,^c^ n (%) **					
Large central metropolitan	22,499 (31.3)	10,525 (58.6)	5,648 (31.4)	3,650 (20.3)	2,676 (14.9)
Large fringe metropolitan	16,470 (22.9)	3,476 (19.3)	4,933 (27.4)	4,373 (24.3)	3,688 (20.5)
Medium metropolitan	14,503 (20.2)	2,337 (13.0)	3,556 (19.8)	3,941 (21.9)	4,669 (26.0)
Small metropolitan	6,527 (9.1)	733 (4.1)	1,371 (7.6)	1,990 (11.1)	2,433 (13.5)
Micropolitan	6,511 (9.1)	565 (3.1)	1,414 (7.9)	2,206 (12.3)	2,326 (12.9)
Noncore	5,389 (7.5)	336 (1.9)	1,058 (5.9)	1,814 (10.1)	2,181 (12.1)
**Census division, n (%) **					
New England	3,357 (4.7)	884 (4.9)	915 (5.1)	851 (4.7)	707 (3.9)
Middle Atlantic	10,047 (14.0)	4,063 (22.6)	2,401 (13.4)	2,080 (11.6)	1,503 (8.4)
East North Central	11,702 (16.3)	2,393 (13.3)	3,310 (18.4)	3,593 (20.0)	2,406 (13.4)
West North Central	5,264 (7.3)	560 (3.1)	1,264 (7.0)	1,897 (10.6)	1,543 (8.6)
South Atlantic	13,528 (18.8)	2,366 (13.2)	3,110 (17.3)	3,215 (17.9)	4,837 (26.9)
East South Central	4,425 (6.2)	592 (3.3)	1,283 (7.1)	1,435 (8.0)	1,115 (6.2)
West South Central	8,085 (11.2)	2,004 (11.2)	2,115 (11.8)	2,003 (11.1)	1,963 (10.9)
Mountain	5,211 (7.2)	914 (5.1)	1,176 (6.5)	1,255 (7.0)	1,866 (10.4)
Pacific	10,280 (14.3)	4,196 (23.3)	2,406 (13.4)	1,645 (9.2)	2,033 (11.3)

Abbreviations: GED, general equivalency diploma; IQR, interquartile range; SD, standard deviation.

a Data source: 5-year estimates from the American Community Survey 2009–2013, including data on self-reported veteran status (13). Concentration of veterans calculated as percentage of veterans among the adult population in each census tract. Concentrations categorized into quartiles: low (0%–6.0%), mid-low (6.0%–8.8%), mid-high (8.8%–11.5%), and high (11.5%–100%). Estimated veteran population: all tracts, 21,080,150; low-concentration census tracts, 2,237,420; mid-low–concentration census tracts, 4,507,889; mid-high–concentration census tracts, 5,991,587; high-concentration census tracts 8,343,254.

b Location data from the Defense Installations Spatial Data Infrastructure Program (19). Distance to nearest military installation calculated as the straight-line distance from the census-tract centroid to the closest boundary of the installation.

c Each census tract classified according to its county location per National Center for Health Statistics’ 2013 Urban–Rural Classification Scheme for Counties (18).

In census tracts with high concentrations of veterans, residents had 0.5 (SD, 0.9; IQR, 0–0.8) supermarkets on average within a 1-mile radius of their residence, while in census tracts with low concentrations of veterans, residents had 3.2 supermarkets (SD, 4.4; IQR, 0.6–3.7) (Table 2). When viewed another way, in 18.7% of high-concentration census tracts and 66.6% of low-concentration census tracts, residents typically lived within 1 mile of at least 1 supermarket. Thus, residents in a high-concentration census tract were 71.9% less likely than residents in a low-concentration census tract to have a supermarket within 1 mile of their residence. The pattern was similar when analyzed by chain and nonchain supermarkets, grocery stores, convenience stores, fast food restaurants, commercial fitness facilities, and parks. Patterns in metropolitan and nonmetropolitan areas were also similar, although the differences in the availability of food and recreational venues across quartiles in nonmetropolitan areas were much smaller. Associations between concentration of veterans and the availability of venues were significant (*P* for all <.001).

**Table 2:** Availability of Food and Physical Activity Venues in Continental US Census Tracts, by Concentration of Veterans, 2009–2013^a^

Venues	All Tracts (N = 71,899)	Census Tract Veteran Concentration Quartile^a^			
		Low (N = 17,972)	Mid–Low (N = 17,980)	Mid–High (N = 17,974)	High (N = 17,973)
**Venue Counts, Mean (SD) [IQR]**					
**Supermarkets**					
All	1.4 (2.7) [0–1.6]	3.2 (4.4) [0.6–3.7]	1.1 (1.7) [0.1–1.6]	0.7 (1.1) [0–1.0]	0.5 (0.9) [0–0.8]
Chain	0.8 (1.2) [0–1.0]	1.5 (1.8) [0.2–2.0]	0.7 (1.0) [0–1.0]	0.5 (0.7) [0–0.7]	0.4 (0.6) [0–0.5]
Nonchain	0.6 (1.7) [0–0.5]	1.7 (3.0) [0–1.9]	0.4 (1.0) [0–0.5]	0.2 (0.6) [0–0.2]	0.1 (0.4) [0–0.1]
**Grocery stores**	4.2 (13.9) [0–2.3]	13.0 (24.5) [0.8–11.6]	2.4 (7.1) [0–2.1]	1.0 (3.7) [0–0.9]	0.6 (2.0) [0–0.5]
**Convenience stores**	4.2 (5.8) [0.3–5.7]	8.7 (8.3) [2.8–11.8]	3.7 (4.4) [0.5–5.6]	2.4 (3.2) [0.1–3.7]	1.8 (2.6) [0.1–2.6]
**Fast food restaurants **					
All	11.5 (24.2) [0.6–13.1]	26.8 (40.7) [6.3–27.9]	9.1 (14.6) [1.0–12.6]	5.7 (9.3) [0.2–8.6]	4.3 (7.5) [0.1–6.3]
Chain	4.6 (6.9) [0.2–6.7]	8.9 (10.3) [2.8–11.6]	4.1 (5.1) [0.4–6.3]	2.9 (4.0) [0.1–4.5]	2.4 (3.6) [0–3.4]
Nonchain	6.9 (18.3) [0.2–6.1]	17.9 (31.8) [2.5–16.9]	5.0 (10.5) [0.4–5.9]	2.8 (6.1) [0.1–3.7]	1.9 (4.5) [0–2.6]
**Commercial fitness facilities**	4.6 (12.3) [0.2–5.1]	9.8 (21.5) [1.8–10.6]	4.0 (7.8) [0.4–5.3]	2.5 (4.8) [0.1–3.5]	1.9 (4.2) [0.1–2.6]
**Parks^b^**	2.3 (2.7) [0.2–3.5]	3.7 (3.2) [1.4–5.2]	2.5 (2.7) [0.4–3.9]	1.7 (2.3) [0.1–2.6]	1.3 (1.9) [0.1–1.7]
**≥1 Venue, n (%)**					
**Supermarkets **					
All	26,466 (36.8)	11,964 (66.6)	6,718 (37.4)	4,418 (24.6)	3,366 (18.7)
Chain	18,842 (26.2)	8,869 (49.4)	4,584 (25.5)	2,988 (16.6)	2,401 (13.4)
Nonchain	11,068 (15.4)	7,290 (40.6)	2,185 (12.2)	1,024 (5.7)	569 (3.2)
**Grocery stores**	26,780 (37.3)	13,032 (72.5)	6,983 (38.8)	4,062 (22.6)	2,703 (15.0)
**Convenience stores**	44,847 (62.4)	15,622 (86.9)	11,935 (66.4)	9,398 (52.3)	7,892 (43.9)
**Fast food restaurants**					
All	50,761 (70.6)	16,493 (91.8)	13,535 (75.3)	11,038 (61.4)	9,695 (53.9)
Chain	45,015 (62.6)	15,603 (86.8)	11,881 (66.1)	9,462 (52.6)	8,069 (44.9)
Nonchain	43,635 (60.7)	15,486 (86.2)	11,742 (65.3)	8,951 (49.8)	7,456 (41.5)
**Commercial fitness facilities**	43,633 (60.7)	14,993 (83.4)	11,740 (65.3)	9,117 (50.7)	7,783 (43.3)
**Parks^b^**	40,438 (56.2)	14,659 (81.6)	10,944 (60.9)	8,125 (45.2)	6,710 (37.3)

Abbreviations: IQR, interquartile range; SD, standard deviation.

a Data source: 5-year estimates from the American Community Survey 2009–2013, including data on self-reported veteran status (13). Neighborhood venue availability was defined as a count of food outlets, commercial fitness facilities, or parks; it represents the average number within a 1-mile radius of the geographic center of all 30 m × 30 m cells (areas) covered by the census tracts with similar veteran concentrations. Concentration of veterans calculated as percentage of veterans among the adult population in each census tract. Concentrations categorized into quartiles: low (0%–6.0%), mid-low (6.0%–8.8%), mid-high (8.8%–11.5%), and high (11.5%–100%). Associations between concentration of veterans and residential environmental feature were tested by using 1-way ANOVA; all found to be statistically significant at *P* < .001.

b Includes local, state, and national parks.

Among all census tracts, location quotients in census tracts with high concentrations of veterans were less than 1.0 for all food outlets: for example, 0.12 for grocery stores, 0.50 for chain supermarkets, and 0.51 for chain fast food restaurants (Figure). Location quotients increased as the concentration of veterans decreased. Location quotients in low-concentration census tracts for all store and restaurant types were greater than 1.0, ranging from 1.97 (chain fast food restaurants) to 3.11 (grocery stores). Results for recreational venues (commercial fitness facilities and parks) were similar. Location quotients in census tracts in nonmetropolitan areas followed similar patterns but tended to be closer to 1 for most measures (Appendix Figure).

**Figure:**
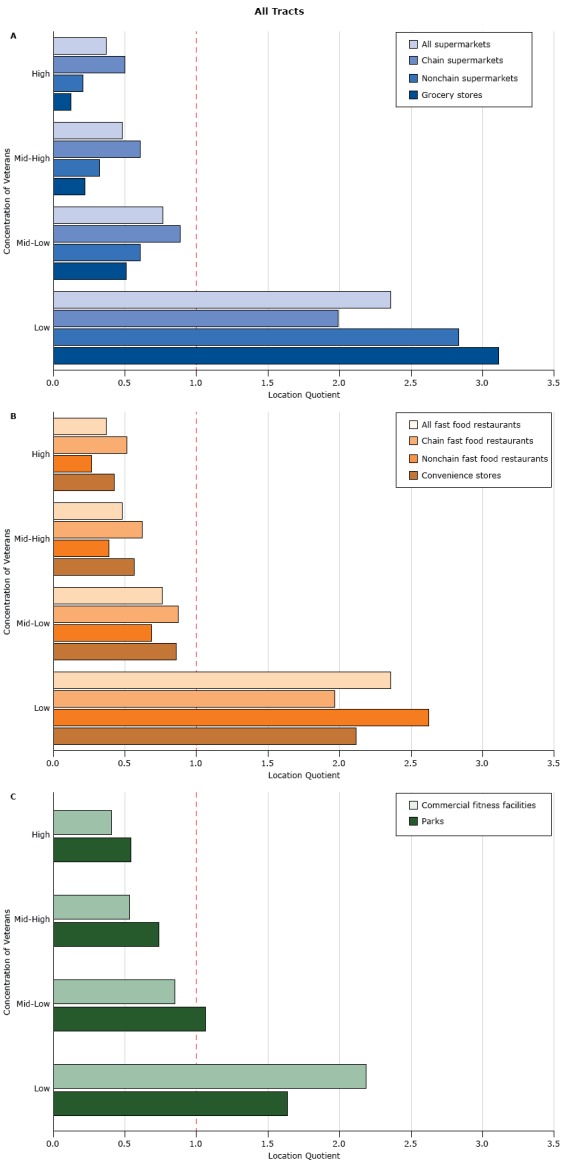
Availability of food and recreational venues in census tracts, by quartile of concentration of veterans (the percentage of veterans among the adult population), relative to all US census tracts, as measured by the location quotient. The greater the location quotient, the greater the availability of a food or recreational venue relative to all locations in a sample. A location quotient equal to 1.0 (indicated by the red dashed line) indicates that availability in sample is equal to availability across all US census tracts: A) supermarkets and grocery stores, B) fast food restaurants and convenience stores, and C) recreational venues. Quartiles of veteran concentration were categorized as low (0%–6.0%), mid-low (6.0%–8.8%), mid-high (8.8%–11.5%), and high (11.5%–100%). **Table:** 

Residential Environmental Attribute	Concentration of Veterans, by Quartile			
	Low	Mid-Low	Mid-High	High
**Grocery stores and supermarkets**				
Grocery stores	3.11	0.51	0.22	0.12
Nonchain supermarkets	2.83	0.60	0.32	0.20
Chain supermarkets	1.99	0.89	0.60	0.50
All supermarkets	2.36	0.76	0.48	0.37
**Fast food restaurants and convenience stores**				
Convenience stores	2.12	0.86	0.57	0.43
Nonchain fast food restaurants	2.62	0.69	0.39	0.27
Chain fast food restaurants	1.97	0.87	0.62	0.51
All fast food restaurants	2.36	0.76	0.48	0.37
**Recreational venues**				
Parks	1.64	1.06	0.73	0.54
Commercial fitness facilities	2.19	0.85	0.53	0.41

In regression analyses of the number of all supermarkets (chain and nonchain combined) within 1 mile of residents (Table 3), Model 1 indicated that supermarket availability varied substantially across geographic areas. Residents of census tracts in large central metropolitan counties (compared with those in less urban counties) and in census tracts with a higher percentage of adults over age 65 years, higher percentages of the population identifying as racial/ethnic minorities, higher percentages of residents with a bachelor’s degree, and lower average income (compared with residents of tracts with fewer adults over age 65, fewer racial/ethnic minority residents, fewer adults with a bachelor’s degree, and higher average income, respectively) had more supermarkets within a 1-mile radius. Model 2 indicated that compared with tracts with low concentrations of veterans and after controlling for other census-tract characteristics, residents of tracts with mid-low, mid-high, and high concentrations of veterans tended to have 0.20 to 0.23 fewer supermarkets within 1 mile (mid-low, β = −0.204, *P* < .001; mid-high, β = −0.205, *P* < .001; high, β = −0.226, *P* < .001). Furthermore, associations of sociodemographic, economic, and geographic characteristics with supermarket availability changed little when variables indicating veteran concentration were added to the model.

**Table 3:** Adjusted Associations Between Concentration of Veterans^a^ and Supermarket Availability in Continental US Census Tracts, 2009–2013^b^

Variable	β (SE) [*P* Value]			
	All Tracts (N = 71,899)		Nonmetropolitan Tracts^c^ (N = 11,900)	
	Model 1	Model 2	Model 1	Model 2
**Concentration of veterans, by quartile^a^**				
Low	Reference		Reference	
Mid-low	NA	−0.204 (0.019) [<.001]	NA	0.047 (0.012) [<.001]
Mid-high	NA	−0.205 (0.021) [<.001]	NA	0.048 (0.013) [<.001]
High	NA	−0.226 (0.023) [<.001]	NA	0.028 (0.013) [.04]
**Percentage of adults aged >65**	0.003 (<0.001) [<.001]	0.006 (<0.001) [<.001]	0.004 (<0.001) [<.001]	0.005 (<0.001) [<.001]
**Race/ethnicity**				
Percentage non-Hispanic white	Reference		Reference	
Percentage non-Hispanic black	0.001 (<0.001) [<.001]	0.001 (<0.001) [.13]	0.002 (<0.001) [<.001]	0.002 (<0.001) [<.001]
Percentage non-Hispanic Asian	0.008 (<0.001) [<.001]	0.006 (<0.001) [<.001]	0.001 (0.002) [.58]	0.002 (0.002) [.41]
Percentage non-Hispanic other	0.005 (0.001) [<.001]	0.005 (0.001) [<.001]	0 (<0.001) [.33]	0 (<0.001) [.42]
Percentage Hispanic	0.001 (<0.001) [.02]	0 (<0.001) [.90]	0.001 (<0.001) [.003]	0.001 (<0.001) [.002]
**Education**				
<High school diploma or GED	Reference		Reference	
High school diploma, no bachelor’s degree	−0.017 (<0.001) [<.001]	−0.015 (<0.001) [<.001]	−0.001 (<0.001) [.03]	−0.001 (<0.001) [<.001]
Bachelor’s degree or more	0.007 (<0.001) [<.001]	0.008 (<0.001) [<.001]	0.002 (<0.001) [.002]	0.002 (<0.001) [.002]
**Median household income, in thousands, $**	−0.007 (<0.001) [<.001]	−0.007 (<0.001) [<.001]	−0.003 (<0.001) [<.001]	−0.003 (<0.001) [<.001]
**Population density, per km^2^, in thousands**	0 (<0.001) [<.001]	0 (<0.001) [<.001]	0 (<0.001) [<.001]	0 (<0.001) [<.001]
**Urbanicity^c^**				
Large central metropolitan	Reference		NA	
Large fringe metropolitan	−0.506 (0.018) [<.001]	−0.502 (0.018) [<.001]		
Medium metropolitan	−0.434 (0.018) [<.001]	−0.417 (0.018) [<.001]		
Small metropolitan	−0.473 (0.024) [<.001]	−0.453 (0.024) [<.001]		
Micropolitan	−0.512 (0.025) [<.001]	−0.487 (0.025) [<.001]	Reference	
Noncore	−0.552 (0.028) [<.001]	−0.531 (0.028) [<.001]	−0.072 (0.006) [<.001]	−0.072 (0.006) [<.001]
**Census division**				
Middle Atlantic	Reference		Reference	
New England	−0.668 (0.032) [<.001]	−0.663 (0.032) [<.001]	0.060 (0.018) [.001]	0.061 (0.018) [.001]
East North Central	−0.518 (0.022) [<.001]	−0.483 (0.022) [<.001]	0.068 (0.013) [<.001]	0.068 (0.013) [<.001]
West North Central	−0.518 (0.028) [<.001]	−0.426 (0.029) [<.001]	0.041 (0.013) [.002]	0.041 (0.013) [.002]
South Atlantic	−0.581 (0.022) [<.001]	−0.542 (0.022) [<.001]	−0.001 (0.014) [.96]	−0.002 (0.014) [.86]
East South Central	−0.633 (0.03) [<.001]	−0.603 (0.03) [<.001]	0 (0.014) [.10]	−0.001 (0.014) [.95]
West South Central	−0.714 (0.025) [<.001]	−0.647 (0.026) [<.001]	0.007 (0.014) [.60]	0.007 (0.014) [.62]
Mountain	−0.603 (0.029) [<.001]	−0.561 (0.029) [<.001]	0.037 (0.016) [.02]	0.039 (0.016) [.01]
Pacific	−0.374 (0.024) [<.001]	−0.348 (0.024) [<.001]	0.053 (0.017) [.002]	0.057 (0.017) [.001]
**Distance to military installation, km^d^**	NA	−0.001 (<0.001) [<.001]	NA	0 (<0.001) [.92]

Abbreviations: GED, general equivalency diploma; NA, not applicable; SE, standard error.

a Data source: 5-year estimates from the American Community Survey 2009–2013, including data on self-reported veteran status (13). Concentration of veterans calculated as percentage of veterans among the adult population in each census tract. Concentrations categorized into quartiles: low (0%–6.0%), mid-low (6.0%–8.8%), mid-high (8.8%–11.5%), and high (11.5%–100%).

b Neighborhood venue availability defined as a count of food outlets, commercial fitness facilities, or parks; it represents the average number within a 1-mile radius of the geographic center of all 30 m × 30 m cells (areas) covered by the census tracts with similar veteran concentrations. Model 1 includes sociodemographic, economic, and geographic characteristics of census tracts. Model 2 adds the concentration of veterans per census tract and a measure of distance to the nearest military installation. *P* values were obtained from ordinary least squares regression models.

c Each census tract classified according to its county location per National Center for Health Statistics’ 2013 Urban–Rural Classification Scheme for Counties (18).

d Location data from the Defense Installations Spatial Data Infrastructure Program (19). Distance to nearest military installation calculated as the straight-line distance from the census-tract centroid to the closest boundary of the installation.

In nonmetropolitan tracts (Table 3), adjusted associations between veteran concentration and supermarket availability were positive, indicating greater supermarket availability with increasing concentration (mid-low, β = 0.047, *P* < .001; mid-high, β = 0.048, *P* < .001; high, β = 0.028, *P* = .04), but coefficients were smaller than those from metropolitan and nonmetropolitan areas combined. Results for all food and recreational variables were similar in direction and significance to those for supermarkets in both metropolitan and nonmetropolitan areas (Appendix Table).

## Discussion

The objective of our study was to characterize the residential environments of the US veteran population with respect to availability of food and recreational venues. We found that areas with high concentrations of veterans were different from areas with low concentrations of veterans. The greater the percentage veterans in an area, the fewer the supermarkets, grocery stores, convenience stores, fast food restaurants, fitness facilities, and parks nearby. We found this relationship in both metropolitan and nonmetropolitan areas. Likewise, the higher the concentration of veterans in a census tract, the fewer the food and recreational venues when compared with the average tract in the continental United States. The associations between the concentration of veterans and the availability of food and recreational venues remained even after we adjusted for a broad set of sociodemographic, economic, and geographic factors with which veteran concentration is correlated and that are themselves known to be associated with residential environmental attributes.

This pattern means that a large percentage (89%) of the veteran population (about 19 million veterans) lives in areas where food outlets that provide variety in healthy food options at the most affordable prices (ie, supermarkets and grocery stores) and places to engage in recreational physical activity are less available than they are for the average adult living in the continental United States. Areas of high concentrations of veterans also have few outlets that predominately offer energy-dense, nutrient-poor foods (ie, convenience stores and fast food restaurants). Although the dearth of convenience stores and fast food restaurants in areas of high concentration of veterans reduces residents’ access to unhealthy foods, supermarkets and grocery stores are still important to facilitate access to healthy foods.

Our findings have implications for veterans’ health. A large body of evidence documents the vulnerability of many veterans to poor health outcomes after military service. After discharge, weight increases, and physical activity declines (20–22). Some evidence suggests that aspects of military service itself may alter food preferences and engender food-related behaviors that predispose some veterans to weight gain (23). Service-connected disability as well as rates of depression and anxiety that are higher in the veteran population than in the civilian population may affect veterans’ food choices and their ability to be physically active (1,2,23,24). Thus, veterans may be particularly vulnerable to weight gain, the chronic conditions associated with weight gain, and their effects on quality of life.

Considering how the environment may contribute to health declines is important. A better understanding of veterans’ residential environments can inform efforts to protect and improve their health. The US Department of Veterans Affairs (VA) has invested heavily in finding ways to help veterans manage their weight. It operates a large-scale nationwide behavioral weight-management program designed to help reduce obesity rates among veterans (25). Information on veterans’ geographic access to food and recreational venues could be incorporated into individual self-management plans and educational content. Successful partnerships between the VA, other federal agencies, and communities such as those targeting homelessness among veterans have demonstrated policy makers’ acknowledgment of and commitment to addressing a broader set of influences on veterans’ health and well-being than is within the reach of individually focused health care services and other benefits provided through the VA. Access to healthy foods at affordable prices and to places for physical activity may be among those influences.

This study has several limitations. Commercial data sources for retail food outlets are prone to error. We carefully cleaned and processed the data to maximize its quality (14). It is reasonable to assume that there is (unobservable) variation in the availability of healthy and unhealthy food products by type of food outlet and variation in the features of recreational venues. If that variation is correlated with the concentration of veterans in census tracts, our findings may not represent true differences in the presence of healthy or unhealthy foods or recreational venues. However, we have no reason to think that such a correlation exists. Furthermore, availability, summarized here as counts within an area, is an important but not the sole determinant of the accessibility of food and recreational venues. The measurement of other accessibility components such as affordability and travel time were beyond the scope of this study. The American Community Survey’s estimates of census-tract population, veteran population, and other characteristics were produced from data collected in 2009–2013. Because census-tract populations are not static, our results reflect population characteristics as they were during 2009–2013 and cannot necessarily be extrapolated to other periods. Finally, our data cannot shed light on the underlying processes that lead to the observed relationships between veterans’ residential locations and the availability of food and recreational venues.

Our study suggests that where veterans live is strongly associated with availability of food outlets that provide healthy (and unhealthy) foods and recreational venues close to home. Given recent recognition of high rates of obesity among veterans and evidence of a link between availability of healthy foods and fast foods and diet and a link between the availability of recreational venues and physical activity, these environmental variations raise questions about their potential effect on veterans’ health. Additional research is needed to address those questions. Furthermore, the findings highlight the potential need for programs aimed at promoting veterans’ health to address or take into account their residential environments.

## Figures and Tables

**Appendix Figure:**
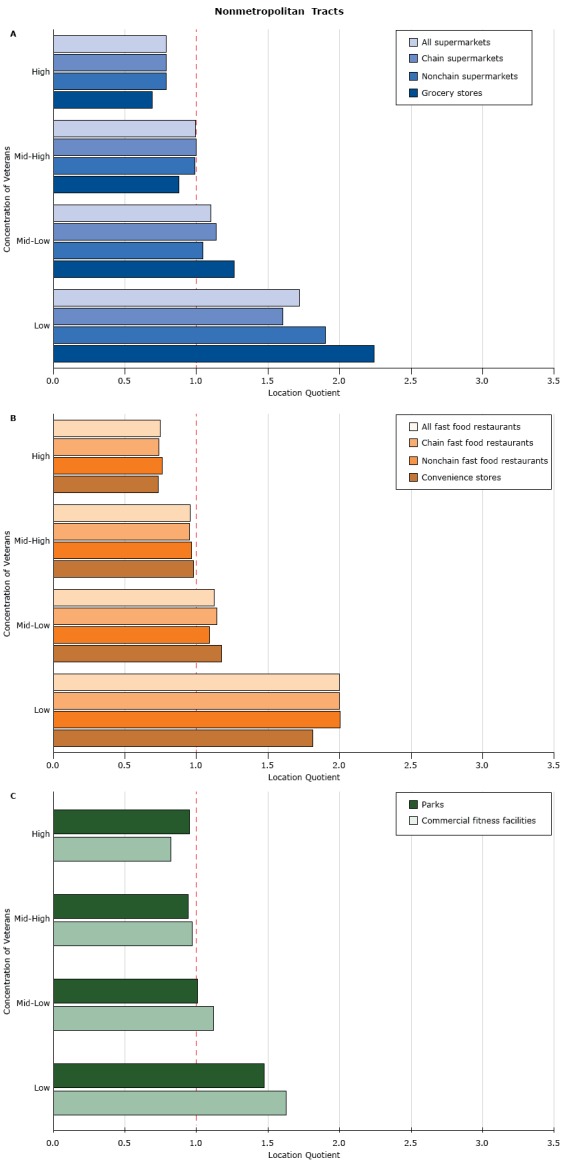
Availability of food outlets and recreational venues in nonmetropolitan census tracts, by quartile of concentrations of veterans (the percentage of veterans among the adult population), relative to all US census tracts, as measured by the location quotient. The greater the location quotient, the greater the availability of a residential environmental attribute relative to all locations in a sample. A location quotient equal to 1.0 (indicated by the red dashed line) indicates that availability in sample is equal to availability across all US census tracts: A) supermarkets and grocery stores, B) fast food restaurants and convenience stores, and C) recreational venues. Quartiles of veteran concentration were categorized as low (0%–6.0%), mid-low (6.0%–8.8%), mid-high (8.8%–11.5%), and high (11.5%–100%). **Table:** 

Residential Environmental Feature	Low	Mid-Low	Mid-High	High
Supermarkets and grocery stores				
Grocery stores	2.24	1.26	0.87	0.69
Nonchain supermarkets	1.90	1.04	0.99	0.79
Chain supermarkets	1.60	1.14	1.00	0.79
All supermarkets	1.72	1.10	0.99	0.79
Fast food restaurants and convenience stores				
Convenience stores	1.81	1.18	0.98	0.73
Nonchain fast food restaurants	2.00	1.09	0.96	0.76
Chain fast food restaurants	2.00	1.14	0.95	0.74
All fast food restaurants	2.00	1.12	0.96	0.75
Recreational venues				
Commercial fitness facilities	1.63	1.12	0.97	0.82
Parks	1.47	1.01	0.94	0.95

## Appendices Supplementary Table and Figure

**Appendix Table:** Adjusted Associations Between Concentration of Veterans and Availability of Various Residential Environmental Attributes in US Continental Census Tracts^a^

Attribute	All Tracts (N = 71,899)			Metropolitan Tracts (N = 59,999)			Nonmetropolitan Tracts (N = 11,900)		
	Low-Mid	High-Mid	High	Low-Mid	High-Mid	High	Low-Mid	High-Mid	High
**Supermarkets**									
All	–0.204	–0.205	–0.226	–0.217	–0.220	–0.242	0.047	0.048	0.028
Chain	–0.085	–0.123	–0.177	–0.093	–0.136	–0.199	0.036	0.034	0.017
Nonchain	–0.119	–0.082	–0.049	–0.124	–0.084	–0.043	0.011	0.014	0.010
**Grocery stores and convenience stores**									
Grocery	–0.957	–0.501	0.080	–0.940	–0.466	0.209^b^	0.045	0.040	0.041
Convenience	–0.847	–1.065	–1.102	–0.860	–1.138	–1.206	0.283	0.287	0.224
**Fast food restaurants**									
All	–1.994	–2.061	–2.079	–2.099	–2.161	–2.144	0.308	0.233	0.111^c^
Chain	–0.453	–0.521	–0.667	–0.491	–0.569	–0.750	0.177	0.119	0.044
Nonchain	–1.541	–1.540	–1.413	–1.608	–1.592	–1.394	0.132	0.114	0.067
**Recreational venues**									
Commercial fitness facilities	–0.785	–1.019	–1.281	–0.885	–1.124	–1.390	0.261	0.215	0.174
Parks	–0.052^d^	–0.317	–0.491	–0.056^e^	–0.348	–0.575	0.123	0.105^f^	0.120

^a ^Percentage of veterans among the adult population in each census tract, determined by using 5-year estimates from the American Community Survey 2009–2013, including data on self-reported veteran status (13). Concentrations categorized into quartiles: low (0%–6.0%), mid-low (6.0%–8.8%), mid-high (8.8%–11.5%), and high (11.5%–100%). Neighborhood venue availability defined as a count of food outlets, commercial fitness facilities, or parks; it represents the average number within a 1-mile radius of the geographic center of all the 30 m × 30 m cells (areas) covered by the census tracts with similar veteran concentrations. *P* values determined by ordinary least-squares regression. Unless otherwise indicated, *P* value is <.001. Reference group for all values is the low quartile.

^b^ *P* = .14.

^c^ *P* = .15.

^d^ *P* = .05.

^e^ *P* = .06.

^f^ *P* = .001.
